# Children's Descriptions of Jubilee Celebrations

**Published:** 1887-07-09

**Authors:** 


### Children's Descriptions of Jubilee Celebrations.

We have had letters from all parts of the country, and it will be interesting, we think, to other children to see how the Jubilee was celebrated in different places far from their own homes.

*Princess Ida at St. Asaph.*—I am going to tell you how the Jubilee of our beloved Queen was spent in St. Asaph, where I live. On the morning of the 21st there was a Jubilee service at the Cathedral, at which a great many people were present. Then at 12 o'clock there was a dinner for all the old men and women, at which two of my sisters assisted. At 1.15 a procession of over 700 children (to each of whom a Jubilee medal was given), the committee, and several ladies, was formed opposite the Deanery, which is about half a mile from the Cathedral, and they marched up to the Cathedral yard, where the Dean planted an American oak in commemoration of the Jubilee; a photograph was taken of the oak tree, with the Dean standing by it, and the committee in a half-circle round. The National Anthem was then sung by the people, and sounded splendid. At 2 o'clock the procession formed again, and proceeded to the National and Infant Schools, where a tea was provided for children of all denominations. Afterwards there were sports in a large field, a band attended, and at 10 o'clock rockets and other fireworks were let off. Bonfires were lit on all the mountains round, that on Moel Famma being especially large. The town was well decorated with flags, arches, etc., and illuminated at night. Altogether, considering the size of the place, I think that the Jubilee was very well celebrated at St. Asaph, and I for one thoroughly enjoyed it, and hope that Her Majesty may live to reign over us for many years.

*Edith at Northampton.*—I think that you might like to know how the Jubilee was spent in Northampton. You might think from the reports that there were nothing but black flags hung out of the windows. In the morning the people marched in procession to church, in number of about thirteen thousand. A dinner was given to the children on Jubilee Day but the old people had theirs on Friday. In the evening there was an ox roasted in the market square, which the people enjoyed seeing very much. The town was brilliantly decorated with flags and flowers, etc. And they had fireworks at night which were very nice.

*Eric at Ardrossan, N.B.*—The way we spent the Jubilee was, we had a lot of bonfires, and we could see about ten of them all round the coast. At 11 o'clock at night we went out in our dressing-gowns to see the bonfires, and we saw lots of rockets going up from the hills. Saltcoats made up a procession, and it went through Ardrossan, and coming back it took the lifeboat out, and all the men in it dressed as they do when they go out to a wreck. In the night some boys put a lot of sticks and rubbish inside a barrel and lit it up, and rolled it about. The town turned out and lit up two bonfires in the street and two on the beach. The streets were crammed with people; and as the clock struck twelve, the whole of them sang at the top of their voices "God save the Queen." Mamma said it sounded very grand, but we were asleep by that time, as we were up a little after six o'clock in the morning.

*Lucky Ellie at Hyde Park.*—I notice the subject this week is how the Jubilee was kept where I live. I am afraid I should take up too much of your time in telling you that; for I want to tell you that I was one of the lucky ones invited to Hyde Park on Wednesday, 22nd ult., and I did so enjoy myself. I had a good look at our Queen, who is my favourite, which I should have told you, if I had written my usual letter last week. I was presented with a handsome medal, and a drinking cup, which I hope to show you some day. There is one thing more I should like to mention before finishing this letter, and that is, that all the nurses at the St. George's Hospital, near Hyde Park Corner, gave us such a cheer, and waved their handkerchiefs as we entered the Park, and perhaps if you think this letter is worth inserting, they will be glad to know that I saw them.

